# Barriers to Palliative Care Integration for Children With Cancer Across Asia Pacific

**DOI:** 10.1200/GO-25-00512

**Published:** 2026-04-15

**Authors:** Andrea Cuviello, Marta Salek, Sri Andini Handayani, Sally Blair, Anjali Chandra, Godwin Job, Bella S. Ehrlich, Lynna Chandra, Ross Drake, Sanjeeva Gunasekera, Mariko Kakazu, Sung Han Kang, Lurdes Maria do R. Leão, Amita Mahajan, Michelle Cristine Miranda, Thida Moe, Mimi Lhamu Mynak, Hoa Thi Kim Nguyen, Eman Rasheed, Sani Sipai, Bounpalisone Souvalansy, Pornpun Sripornsawan, Teresa Shu Zhen Tan, Kok Hoi Teh, Nobuyuki Yotani, Chong Lee Ai, Poonam Bagai, Ong Gek Bee, Shella Bravo, Siti Nur Hanim Buang, Huyen Thi Thanh Bui, Tashi Choden, Bishnu Rath Giri, Dylan E. Graetz, Haresh Gupta, Chong Poh Heng, Erica C. Kaye, Chusana Khaiman, Aye Aye Khaing, Min Sun Kim, Nia Kurniati, Stephen Laughton, Ratha Mlis, Lan Bui Ngoc, Ximena Garcia Quintero, Diana Rios, Milena Maria Lay Dos Santos, Sudhir Sapkota, Teny Tjitra Sari, Krishna Sagar Sharma, Rojim Sorrosa, Sommaphun Tabjaroen, Rina Wahyuni, Kazuyo Watanabe, Su Yandana, Wynn Yi Yi, Maziya Abbas Zaki, Meenakshi Devidas, Justin N. Baker, Michael J. McNeil, Asya Agulnik, Catherine G. Lam

**Affiliations:** ^1^ Phoenix Children’s Hospital, Phoenix, AZ; ^2^ St Jude Children’s Research Hospital, Memphis, TN; ^3^ Stanford University School of Medicine, Palo Alto, CA; ^4^ Boston Children’s Hospital, Boston, MA; ^5^ Rachel House (Yayasan Rumah Rachel), Jakarta, Indonesia; ^6^ Queensland Children’s Hospital, Brisbane, Australia; ^7^ National Cancer Institute, Maharagama, Sri Lanka; ^8^ Japan Heart Medical Center, Udong, Cambodia; ^9^ Asan Medical Center Children's Hospital, Seoul, South Korea; ^10^ National Hospital Guido Valadares, Díli, Timor-Leste; ^11^ Indraprastha Apollo Hospitals Delhi, Delhi, India; ^12^ Southern Philippines Medical Center, Davao City, Philippines; ^13^ Yangon Children's Hospital, Yangon, Myanmar; ^14^ Jigme Dorji Wangchuck National Referral Hospital, Ministry of Health, Thimphu, Bhutan; ^15^ Hue Central Hospital, Hue, Vietnam; ^16^ Hulhumalé Hospital, Hulhumalé, Maldives; ^17^ Kanti Children's Hospital, Kathmandu, Nepal; ^18^ Children's Hospital, Vientiane, Laos; ^19^ Prince of Songkla University, Hat Yai, Thailand; ^20^ National University Children's Medical Institute, Singapore, Singapore; ^21^ Hospital Tunku Azizah, Kuala Lumpur, Malaysia; ^22^ National Center for Child Health and Development, Tokyo, Japan; ^23^ University Malaya, Kuala Lumpur, Malaysia; ^24^ CanKids KidsCan, Delhi, India; ^25^ Sabah Women and Children Hospital, Sabah, Malaysia; ^26^ KK Women's and Children's Hospital, Singapore, Singapore; ^27^ VinUniversity Hanoi, Ha Noi, Vietnam; ^28^ WHO Regional Office for Southeast Asia, New Delhi, India; ^29^ HCA Hospice Care, Singapore, Singapore; ^30^ Chulalongkorn University, Bangkok, Thailand; ^31^ University of Medicine, Maharagama, Myanmar; ^32^ Seoul National University Hospital, Seoul, Republic of Korea; ^33^ Cipto Mangunkusumo Hospital, Jakarta, Indonesia; ^34^ Starship Blood and Cancer Center, Auckland, New Zealand; ^35^ Calmette Hospital, Phnom Penh, Cambodia; ^36^ Vietnam National Children's Hospital, Hanoi, Vietnam; ^37^ B.P. Koirala Memorial Cancer Hospital, Chitwan, Nepal; ^38^ Thai Pediatric Oncology Group, Bangkok, Thailand; ^39^ Asian Children's Care League, Tokyo, Japan

## Abstract

**PURPOSE:**

Early integration of palliative care (PC) for children with cancer is vital to improved patient outcomes. Despite this, access to PC services for children in Asia Pacific (AP) is sparce. We aimed to explore potential barriers to PC provision in childhood cancer care across AP.

**METHODS:**

The Assessing Doctors' Attitudes on Palliative Treatment survey was revised for cultural context, translated into six languages, and distributed electronically to physicians who care for children with cancer. Descriptive statistics were used to summarize data. Secondary analyses used the Pearson chi-square test or Fisher exact test to examine associations between prior PC training and physician subspecialty with reported barriers. The McNemar test was applied to evaluate differences between actual versus ideal timing of PC consultations. Analysis of variance was conducted to compare mean values for perceived barriers across country income levels.

**RESULTS:**

Six hundred twenty-one physicians from 18 countries participated, with an overall response rate of 27% (621/2,305) and a median country response rate of 30% (range, 11%-85%). Most respondents (n = 366; 59%) believed PC should be consulted at diagnosis, but only 18% (n = 117) stated that this occurred in their clinical setting (*P* < .001). The most highly ranked barriers to PC provision included limited physician knowledge (n = 511; 82%), lack of national health policy/advocacy (n = 494; 79%), lack of PC-trained clinicians (n = 492; 79%), lack of home-based services (n = 487; 78%), and physician discomfort in discussing PC (n = 483; 78%). Physicians practicing in lower-middle–income countries rated these barriers more significantly.

**CONCLUSION:**

Physicians report a discrepancy between ideal and actual timing of PC integration and identify several barriers to PC provision. Study findings will inform capacity building, education, and advocacy initiatives to improve the timing and quality of PC delivery in the region.

## INTRODUCTION

The integration of palliative care (PC) across the continuum of childhood cancer care refers to the involvement of PC specialists and services throughout the entire course of a child's illness. This approach has been shown to improve patient outcomes, including quality of life, family-clinician communication, and end-of-life and bereavement care.^1-6^ Despite this evidence, PC is not accessible to all children and families facing cancer. Barriers to PC provision include lack of physician understanding and awareness of PC, lack of PC resources, and perceived patient/family hesitancy.^7-9^ Research related to PC in pediatric cancer care delivery has been primarily conducted in high-income countries, and translation of this evidence across cultures and resource-varied settings is unclear.

**CONTEXT**

**Key Objective**
We used the Assessing Doctors' Attitudes on Palliative Treatment Survey to explore ideal versus actual timing for palliative care (PC) consultation and the potential barriers to PC provision in childhood cancer care across Asia Pacific.
**Knowledge Generated**
Over 600 physicians responded, representing 18 countries, and reported a discrepancy between ideal and actual timing of PC integration, with PC consultation often occurring too late. The most highly ranked barriers to PC provision included limited physician knowledge, lack of national health policy/advocacy, lack of PC-trained clinicians, lack of home-based services, and physician discomfort in discussing PC with patients/families, with a stronger emphasis placed on these barriers by physicians practicing in lower-middle–income countries.
**Relevance**
Findings from this study will inform capacity building, education, and advocacy initiatives to improve the timing and quality of PC delivery in the region.

Globally, there are an estimated 20 million children in need of PC and approximately 30% of this patient burden exists in Southeast Asia and the Western Pacific, collectively contributing to the Asia Pacific (AP) region.^10^ Within AP, there are an estimated 72,000 children diagnosed with cancer each year.^10^ As a large and heterogeneous region, there are many cultures, languages, and mechanisms for health care delivery for children with cancer, including the provision of PC services.^10-12^ As a result, disparities exist related to treatment of childhood cancer and pediatric PC access across and within individual countries.^11^ Efforts to improve access to supportive care services in oncology, including PC, are of high priority within AP.^6^

The Assessing Doctor's Attitudes on Palliative Treatment (ADAPT) study was designed to explore physician's knowledge, attitudes, and barriers to PC integration for children with cancer.^13,14^ Initially designed for distribution in Eastern Europe and Central Asia (Eurasia), the survey has been subsequently adapted in other world regions, resulting in regional research, education, and advocacy efforts to improve PC access.^15-18^ To better understand approaches to PC provision for children receiving cancer care in AP, this study aimed to explore ideal versus actual timing of PC consultation in current clinical practice and assess barriers to PC integration in the region, using the previously validated ADAPT survey.

## METHODS

### Instrument Development

The original ADAPT survey was developed by an international panel of pediatric oncology and PC experts.^13,14,19-21^ Adaptation of this survey to reflect cultural sensitivity and content validity for the AP region was completed by a nine-member expert panel as previously described,^22^ resulting in the addition of nine questions while maintaining integrity with prior survey iterations. The final ADAPT-AP survey consisted of 74 items, including 69 quantitative (using either a five-point Likert scale or multiple-choice format) and five qualitative items soliciting free-text responses (Data Supplement, Fig S1).

The lead institutional review board for this study was St Jude Children's Research Hospital in Memphis, Tennessee, which approved this study as exempt research. All survey data were anonymized. Additional approvals from participating institutions were obtained as needed and are described elsewhere.^22^ Voluntary completion of this survey was considered consent to participation in the study. The study followed the American Association for Public Opinion Research reporting guideline.^23^

### Instrument Distribution Strategy

The survey was distributed via the Qualtrics electronic software platform^24^ to physicians caring for children with cancer (ie, general pediatricians and subspecialists) in 18 participating counties: Bhutan, Cambodia, India, Indonesia, Japan, Korea, Laos, Malaysia, Maldives, Myanmar, Nepal, New Zealand, the Philippines, Singapore, Sri Lanka, Thailand, Timor-Leste, and Vietnam. Research collaborators were identified from each country through the St Jude Global Alliance, an international network of over 300 medical institutions and foundations from more than 80 countries dedicated to improving outcomes for children facing catastrophic illness globally.^25^ Routine meetings with country collaborators facilitated identification of eligible participants, translation needs, and the development of strategies for survey engagement and distribution.^22^ The survey was distributed asynchronously in cohorts from February 2022 through February 2024 and was open for 12 weeks in each country. Country teams received updates on the number of completed surveys every 1-2 weeks during the data collection period. Local teams then encouraged study participation by sending reminders via e-mail or instant messaging services. Only responses from physicians who completed all survey items were included in data analysis. A previously published commentary summarizes the challenges encountered in adapting and translating the survey instrument, obtaining local study approvals, and implementing and distributing the study across the AP region.^22^

### Statistical Analysis

Descriptive statistics were used to summarize region- and country-specific demographic data.^26^ Secondary analysis of responses to questions using five-point Likert scales were condensed into three categories (extremely or somewhat important, neither important nor unimportant, and somewhat or extremely unimportant) to compare associations with demographic variables using the Pearson chi-square test or Fisher exact test. The McNemar test was used to evaluate differences between related to actual versus ideal timing of PC consultations. Mean values for perceived barriers across country income levels were compared using analysis of variance.

Statistical significance was determined using a two-tailed *P* value threshold of <.05. All summaries and analyses were performed using SAS version 9.4 (SAS Institute Inc, Phoenix, AZ).^27^

### Qualitative Analysis

Written, free-text responses were translated into English (if applicable) by study team members (A.C. and M.S.) using online software. The codebook was developed using deductive codes from qualitative analysis approaches used in previous ADAPT studies.^14,28^ Additional novel codes inductively identified through analysis of free-text responses were included in the final codebook (Data Supplement, Table S1), which was applied across transcripts by two study team members (A.C. and M.S.) Discrepancies were resolved by consensus with a third-party adjudicator (C.L.) when necessary. Each free-text response served as a unit of analysis. Thematic content analysis was conducted to examine differences between ideal versus actual timing of PC involvement, as well as to identify common barriers to PC integration. Qualitative data were organized using MAXQDA 2024 software (WERBI, Berlin, Germany).^29^

## RESULTS

### Participant Demographics

The ADAPT-AP survey included participation 621 physicians, representing 18 countries. Countries represented lower-middle–income (LMIC; n = 10), upper-middle–income (UMIC; n = 4), and high-income (HIC; n = 4) countries as defined by the World Bank.^30^ The median country response rate was 30% (range, 11%-85%; Data Supplement, Table S2) and the overall response rate was 27% (621/2,305; Table 1). Of the 621 respondents, 60% (n = 373) identified as female, and 57% (n = 352) were age 35-50 years, with most having over 11 years of clinical experience (69%; n = 432; Table 1). Only one third of respondents (35%; n = 220) reported having received PC training. Many identified as pediatric hematology/oncology specialists (43%; n = 266) or general pediatric practitioners (25%; n = 156), while the remaining third (32%; n = 199) represented a range of other pediatric subspecialties. Most respondents reported their local institutions offered PC services (73%; n = 453); however, only 57% (n = 353) had access to a pediatric PC clinician, most of whom were physicians (97%; n = 341). Almost all respondents (90%; n = 556) had at least one patient die under their care within the last year.

**TABLE 1:** Demographic Characteristics of Respondents Participating in the Assessing Doctors' Attitudes on Palliative Treatment Survey in Asia Pacific

Demographic Characteristic	Respondents, No. (%)
Country	
Bhutan	18 (2.9)
Cambodia	10 (1.6)
India	103 (16.6)
Indonesia	33 (5.3)
Japan	50 (8.0)
Laos	42 (6.8)
Malaysia	49 (7.9)
Maldives	4 (0.6)
Myanmar	46 (7.4)
Nepal	25 (4.0)
New Zealand	18 (2.9)
The Philippines	56 (9.0)
Republic of Korea	12 (1.9)
Singapore	44 (7.1)
Sri Lanka	11 (1.8)
Thailand	25 (4.0)
Timor-Leste	6 (1.0)
Vietnam	69 (11.1)
Age	
<35 years	137 (22.0)
≥35 years	484 (78.0)
Sex	
Female	373 (60.1)
Male	246 (39.6)
Prefer not to disclose	2 (0.3)
Primary medical specialty	
General pediatrics	156 (25.1)
Pediatric hematology/oncology	265 (42.7)
Pediatric PC	17 (2.7)
Other^a^	183 (29.5)
Primary institution	
General hospital	312 (50.2)
Children's hospital	205 (33.0)
Cancer hospital	79 (12.7)
Other	25 (4.1)
Years of experience	
0-10 years	189 (30.4)
≥11 years	432 (69.6)
Trained in PC	
Yes	220 (35.3)
No	401 (64.7)
Access to PC consultation	
Yes	452 (72.8)
No	169 (27.2)
Access to pediatric PC expert	
Yes	352 (56.7)
No	269 (43.3)
Patients who died during care in previous year, No.	
0	65 (10.5)
1-5	322 (51.9)
≥6	234 (37.6)

NOTE. Other primary medical specialties includes pediatric or adult anesthesiology, pediatric or adult surgery, adult or general PC, adult hematology and/or oncology, general internal medicine/family medicine, pediatric intensive care, pediatric ophthalmology, pediatric pulmonology, pediatric infectious diseases, adult, pediatric, or general radiation oncology, genetics, neurosurgery, ocular oncology, and pediatric and/or adult orthopedic oncology.

Abbreviation: PC, palliative care.

a Pediatric anesthesiology, Pediatric surgery, Pediatric intensive care, Adult PC, General PC, General internal medicine/family medicine, Adult hematology and/or oncology, Adult anesthesiology, Adult surgery and Adult intensive care.

### Timing of PC Consultation

**FIG 1:**
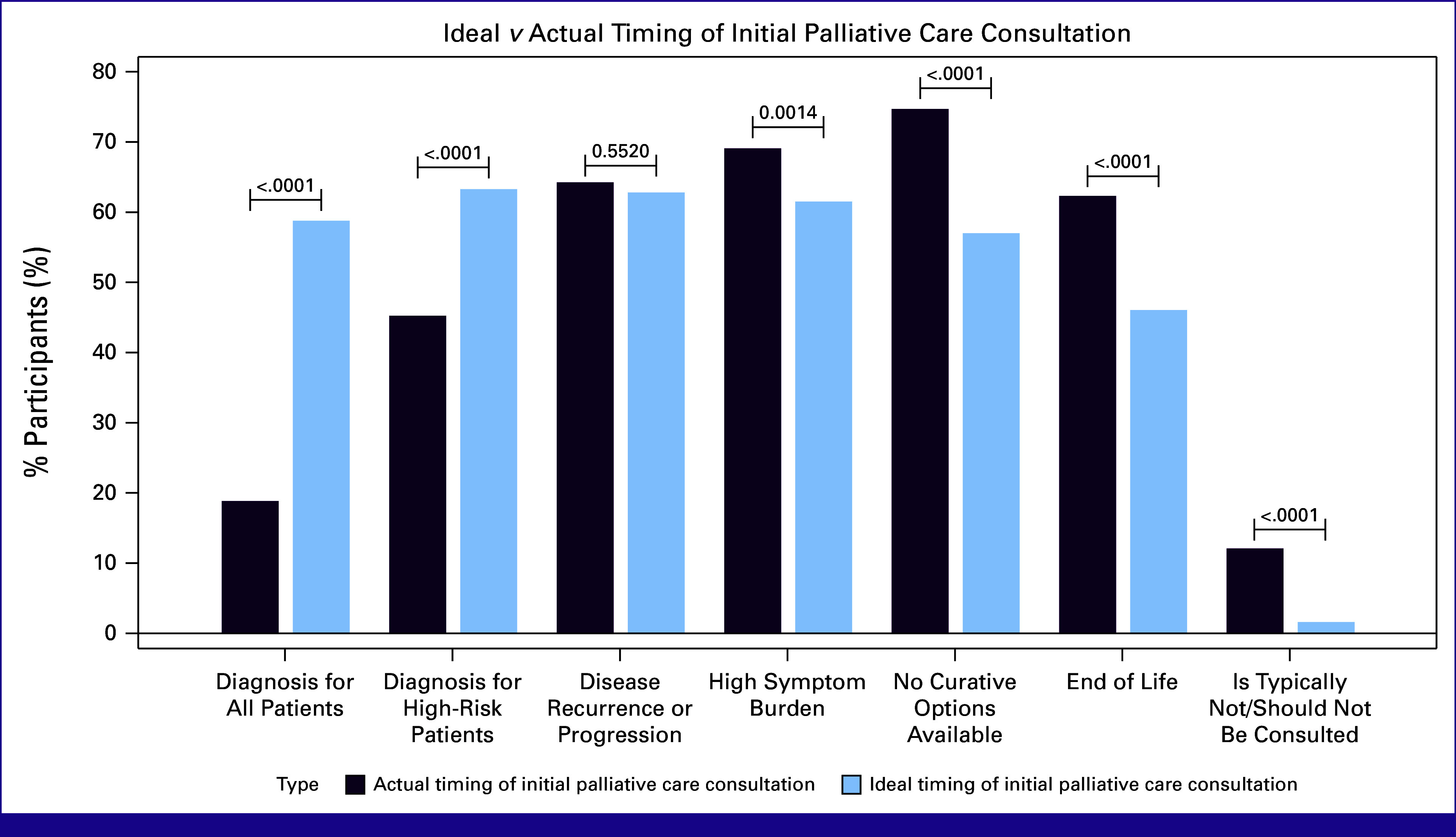
Ideal versus actual timing of initial PC consultation. Results of 621 physician respondents to the multiple-choice questions regarding when initial PC consultation occurs for a child with cancer in their practice setting and when they believe the ideal timing for PC consultation should be. Participants were able to choose multiple options as applicable. PC, palliative care.

Most participants (75%; n = 464) reported that, in their clinical setting, the PC team is consulted for children diagnosed with cancer when no curative treatment options remain (Fig 1). Additional time points for PC involvement included at the time of complex or high symptom burden (69%; n = 429), disease relapse or progression (64%; n = 399), or at end-of-life (62%; n = 387). 12% (n = 75) reported that PC is never consulted due to lack of resource availability. Physicians working at children's hospitals were more likely than those working at general or cancer hospitals to report that PC is consulted at diagnosis for children at high risk of relapse or experiencing disease progression (children's hospital, n = 113/205 [55%]; general hospital, n = 129/312 [41%]; cancer hospital, n = 32/79 [41%]; *P* < .01) and at the time of relapse (children's hospital, n = 146/205 [71%]; general hospital, n = 189/312 [61%]; cancer hospital, n = 54/79 [68%]; *P* < .01; Data Supplement, Table S3).

When asked about the ideal timing for PC consultation, over half of the respondents (59%; n = 366) identified that PC should be involved at diagnosis for all children diagnosed with cancer; however, only 19% (n = 117) reported that this occurred in their setting (*P* < .001; Fig 1). Nearly two thirds (63%; n = 393) reported PC should be involved at diagnosis for all children with high-risk disease. However, in practice, this occurred only 45% of the time (n = 282; *P* < .001). Similarly, 38% of respondents (n = 236) reported that PC consultations often or always occurred too late in the care of a child with cancer. Very few participants (2%; n = 10) believed that PC should never be consulted, even if available. No statistically significant differences were observed in responses regarding the ideal timing of PC consultation based on the respondent's primary institution.

### Barriers to PC Provision

Of the 15 proposed barriers, nearly all were recognized by at least half of the respondents as being somewhat or extremely important in hindering the provision of PC (Table 2). The most frequently identified barriers included limited physician PC knowledge (82%; n = 511), lack of national PC-focused health policy and advocacy efforts (80%; n = 494), lack of PC-trained clinicians (79%; n = 492), lack of home-based services (78%; n = 487), and physician discomfort in discussing PC with patients/families (78%; n = 483).

**TABLE 2:** Barriers to Early PC Integration

Barrier	Respondents Indicating Somewhat and/or Extreme Importance (N = 621), %	Mean Score (SD)
Limited physician knowledge on the role of PC	82.3	3.98 (0.95)
Physician discomfort in raising the topic of PC with families	77.6	3.85 (0.93)
Physician desire to maintain hope	64.1	3.59 (0.96)
Uncertainty about patient prognosis	61.5	3.53 (1.00)
Family resistance to involvement of PC	75.5	3.87 (0.98)
Time constraints of pediatric oncologists during consultation	65.2	3.60 (1.03)
Lack of home-based services	78.4	3.96 (1.03)
Limited access to opioids	55.6	3.31 (1.33)
Limited access to PC specialists or services	74.9	3.86 (1.10)
Lack of national health care policies and advocacy efforts on pediatric PC	79.6	3.98 (1.01)
Lack of pediatric PC training in the country	79.1	3.96 (1.01)
Cost of PC consultation and treatment	52.5	3.28 (1.29)
Cultural differences between patients/families and physicians	56.2	3.38 (1.09)
Cultural perception or local general understanding of pediatric PC	68.4	3.70 (1.00)
Differences in languages between patients/families and physicians	47.2	3.22 (1.16)

Abbreviation: PC, palliative care.

Variations in the perceived importance of specific barriers to PC services were significantly associated with physician specialty and prior PC training. PC physicians were less likely than general pediatricians, pediatric hematologists-oncologists, and physicians from other disciplines to report discomfort (*P* < .01), prognostic uncertainty (*P* < .05), limited access to opioids (*P* < .01), lack of PC training (*P* < .05), cost of PC (*P* < .001), cultural differences between patient/family and physician (*P* < .05), cultural perception of PC (*P* < .05), or differences in language (*P* < .05) as important barriers to PC provision (Data Supplement, Table S4). Respondents with previous PC training were less likely than physicians without previous PC training to identify limited access to opioids (*P* < .05), lack of national PC-focused health policy and advocacy efforts (*P* < .005), lack of PC training (*P* < .005), cost of PC (*P* < .0001), cultural difference between patient/family and physician (*P* < .01), or cultural perception of PC (*P* < .05) as important barriers to the provision of PC (Data Supplement, Table S5).

**FIG 2:**
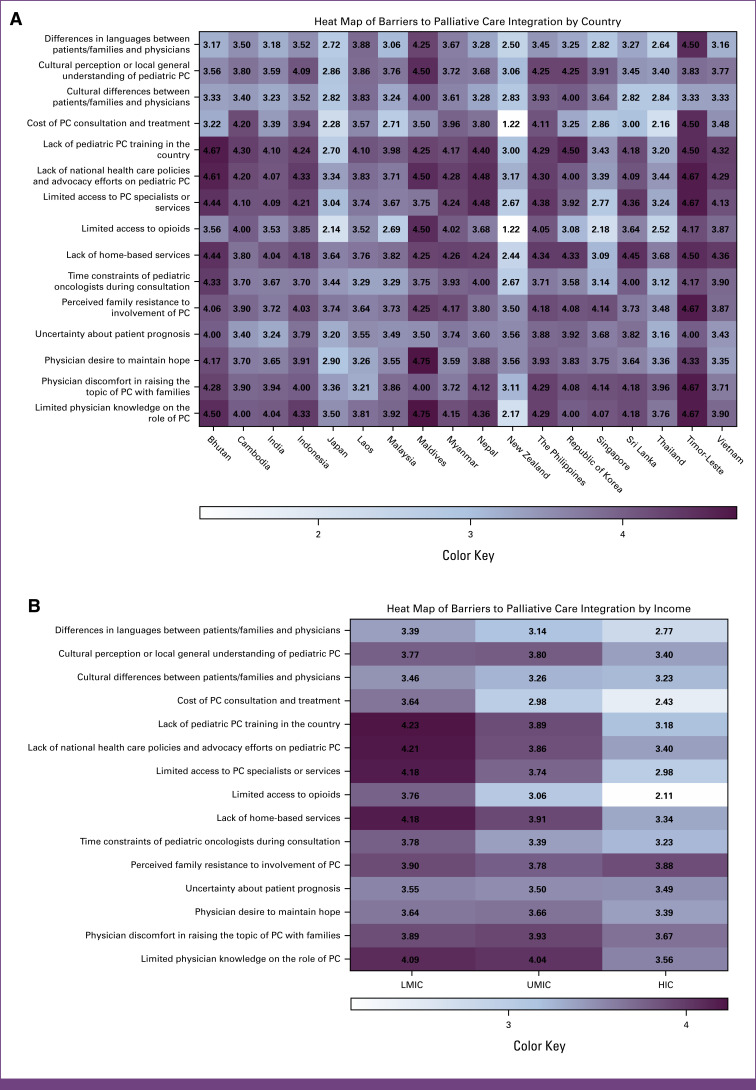
Mean score of significant barriers to PC integration by country and income level. (A) The heatmap shows country-specific reporting of each potential barrier on the early integration of PC for children with cancer, as well as the barriers' perceived importance as identified by participants (n = 622). The five-point Likert scales were converted to numeric values, with one indicating extremely unimportant and five indicating extremely important barriers. The numbers represented in the figure demonstrate the mean score within each country for each barrier. The darker the color, the higher the numeric value. (B) The heatmap shows, by aggregated countries' scores per World Bank income level, each potential barrier on the early integration of pediatric PC for children with cancer, as well as the barriers' perceived importance as identified by participants (n = 622). The responses are shown as lower middle income, upper middle income, or high income. The five-point Likert scales were converted to numeric values, with one indicating extremely unimportant and five indicating extremely important barriers. The numbers represented in the figure demonstrate the mean score within each income group for each barrier. The darker the color, the higher the numeric value. HIC, high-income country; LMIC, lower-middle–income country; PC, palliative care; UMIC, upper-middle–income country.

Differences in the perceived importance of barriers to PC integration were observed across countries and income levels. Figures 2A and 2B present heatmaps of the mean importance score for each barrier, with darker-colored boxes indicating barriers rated as more significant by participants. Quantitative questions related to importance of barriers to PC integration were assessed using a five-point Likert scale, with answers ranging from one (extremely unimportant) and five (extremely important). The mean score of each included barrier was lowest among participating HIC, while responses from LMIC and UMIC were often similar (Table 3). The most pronounced differences were observed for limited PC knowledge (*P* < .0001), physician time constraints (*P* < .0001), lack of home-based services (*P* < .0001), limited access to opioids (*P* < .0001), limited access to PC specialists or services (*P* < .0001), lack of national PC-focused health policy and advocacy efforts (*P* < .0001), lack of PC training (*P* < .0001), cost of PC (*P* < .0001), and differences in language (*P* < .0001; Fig 2B).

**TABLE 3:** Mean Scores by World Bank Status

Variable	Mean Score (SD)			*P*
	LMIC (n = 386)	UMIC (n = 111)	HIC (n = 124)	
Limited physician knowledge on the role of PC	4.09 (0.90)	4.04 (0.84)	3.56 (1.10)	<.0001
Physician discomfort in raising the topic of PC with families	3.89 (0.91)	3.93 (0.86)	3.67 (1.00)	.0456
Physician desire to maintain hope	3.64 (0.96)	3.66 (0.84)	3.39 (1.04)	.0289
Uncertainty about patient prognosis	3.55 (1.04)	3.50 (0.95)	3.49 (0.94)	.8061
Family resistance to involvement of PC	3.90 (1.00)	3.78 (0.98)	3.88 (0.92)	.5526
Time constraints of pediatric oncologists during consultation	3.78 (0.96)	3.39 (1.11)	3.23 (1.04)	<.0001
Lack of home-based services	4.18 (0.91)	3.91 (0.99)	3.34 (1.17)	<.0001
Limited access to opioids	3.76 (1.11)	3.06(1.29)	2.11 (1.20)	<.0001
Limited access to PC specialists or services	4.18 (0.86)	3.74 (1.07)	2.98 (1.27)	<.0001
Lack of national health care policies and advocacy efforts on pediatric PC	4.21 (0.89)	3.86 (1.06)	3.40 (1.07)	<.0001
Lack of pediatric PC training in the country	4.23 (0.80)	3.89 (0.97)	3.18 (1.21)	<.0001
Cost of PC consultation and treatment	3.64 (1.15)	2.98 (1.29)	2.43 (1.24)	<.0001
Cultural differences between patients/families and physicians	3.46 (1.06)	3.26 (1.14)	3.23 (1.14)	.0488
Cultural perception or local general understanding of pediatric PC	3.77 (0.95)	3.80 (1.00)	3.40 (1.11)	.0006
Differences in languages between patients/families and physicians	3.39 (1.12)	3.14 (1.17)	2.77 (1.18)	<.0001

Abbreviations: HIC, high-income country; LMIC, lower-middle–income country; PC, palliative care; SD, standard deviation; UMIC, upper-middle–income country.

Respondents highlighted challenges in accessing PC services as well as distinct attitudes and perceptions from societal, caregiver, and physician perspectives when reflecting on actual versus ideal timing of PC consultations in their settings. One participant shared, “There is a difference because of inadequate human resources to provide early PC to patients and PC is still considered end-of-life care.” Another respondent stated, “[There is a] lack of awareness and prejudice among medical staff, guardians, and patients regarding PC.”

Identifying top priority areas for PC development, qualitative responses aligned with quantitative findings regarding reported barriers to PC integration. Specifically, priorities centered on increasing physician awareness and understanding of PC, increasing the number of trained PC professionals, and strengthening advocacy and funding support. As one participant reported, “First of all, we need to expand our [PC] knowledge to other doctors, [so they can] understand [PC] deeply. We will then be able to explain to the child's family [and help them] understand [PC], be ready to listen, and ready to devote time, energy, and effort to [help] the child to have memories and happiness until their last breath.”

## DISCUSSION

This study reports physician approaches to PC involvement and barriers to the provision of PC services for children with cancer in 18 countries across AP. Despite established evidence for the benefits of PC integration in childhood cancer care, physicians in AP report discrepancies between ideal and actual timing of PC integration. Study findings demonstrate that physicians believe that PC should be integrated earlier in childhood cancer care, with consultation often occurring too late in a child's treatment course (ie, in the setting of exhausted options for curative-intent therapy or at end of life). Additionally, participants identified several barriers to PC provision, including limited physician knowledge and discomfort recommending PC, lack of PC resources, limited capacity for home-based services, and lack of national health policy and advocacy efforts supporting PC development. Respondents from LMIC rated all barriers as more significant to those from UMIC and HIC, underscoring variations between countries with differing health care resources and infrastructure.

In comparison with previously published ADAPT results in Eurasia, Latin America, and Western Europe, our findings are congruent, with all studies highlighting the perceived need for earlier integration of PC for children receiving cancer treatment.^14,18,28^ The top barriers to PC provision identified by physicians were similar across all regions, including physician PC knowledge, lack of access to home-based services, and limited access to PC trained multidisciplinary clinicians. This demonstrates that despite differences in language, culture, and health care infrastructure, global challenges to PC integration remain in childhood cancer. These study findings identify opportunities for future interventions to address identified barriers and improve childhood cancer care. Potential interventions include increased PC education and training and development of home-based services. Regional and cross-regional collaborations can help clinicians, advocates, program planners, and other implementers learn from shared experiences as context-specific strategies are developed and deployed. Distinct barriers reported by physicians in AP include the need for national policy and advocacy support for PC and limitations to opioid access, with both of these barriers rooted in challenges at a health systems level that warrant multisectoral solutions, beyond what can be navigated by physicians alone^31^ Moreover, these barriers were reported as more important or burdensome by physicians practicing in LMIC (*v* UMIC and HIC), reinforcing the need for increased and integrated national and systems level support to clinical teams practicing in lower-resourced settings.

Additionally, perceived barriers to PC integration varied between physicians with or without PC expertise, consistent with prior ADAPT studies. For example, PC physicians reported lower scores (corresponding to lower level of concern) related to perceived barriers, including opioid access or cultural stigma surrounding PC when compared with physicians without PC training. When interpreting these results, it should be noted that participants with prior PC training reported completing a wide range of training programs, from brief courses to an extensive master's certifications, resulting in varying exposure to PC concepts. Nonetheless, improved access to PC education and training may help mitigate these perceived barriers. PC-trained physicians also did not report that perceived patient/family resistance to PC integration was a significant barrier to care provision. Research conducted in HIC has demonstrated that patients and families are often open to PC involvement and prefer early integration.^32^ Study findings may reflect a physician's own discomfort or resistance to PC integration rather than the patient/family perspective. Future research examining patient and family perceptions of PC in AP are critical to the development of interventions that are culturally and contextually relevant.

Although more than half of the physicians in this study reported access to PC services (n = 353/621; 57%), respondents working in two countries (Maldives and Timor-Leste) reported no local PC services, accentuating the regional heterogeneity in PC access in childhood cancer care. These findings are similar to those reported in Eurasia (n = 230/424; 54%)^13^ and slightly lower than those reported in Latin America (n = 556/831; 67%),^28^ highlighting the global need for increased PC resources. Specifically, physicians identified time constraints and limited local PC workforce as contributors, and these limitations appear to be pervasive, regardless of country income level. Research conducted in HIC has demonstrated that late integration or underutilization of PC was related to limitations in accessible PC-trained providers.^9,31,33^ Efforts to increase the PC education and training of health care professionals is a vital element to increase access to PC, and ADAPT work in other regions has inspired multidisciplinary Education in Palliative and End-of-Life Care-Pediatrics training seminars.^16,21^ The findings from ADAPT-AP will also inform country- and region-level reports that include clinical and policy recommendations. Similar reports generated from ADAPT studies conducted in Eastern Europe/Central Asia and Latin America have been successful evidence-based advocacy tools that bridge the gap between research, policy, and health care delivery.^15,34^ Capacity building and growth of the PC workforce are also necessary in promoting earlier PC integration and were reported as top priorities for bridging the gap between actual and ideal timing for PC integration in childhood cancer care in this study.

Several limitations to this study exist. First, survey response rates varied between countries, and the overall response rate was low despite comprehensive country-specific approaches to solicit physician engagement. This was in part due to the differences in physician population sizes and limited protected time for and/or interest in participating in a survey-based study in the setting of high clinical burdens. Notably, AP has a lower number of physicians per capita relative to the regions examined in prior ADAPT analyses (Eurasia and Latin America).^22,35^ Efforts to encourage survey participation included making the survey accessible to regional participants in AP by translating the survey into six languages in addition to English.^22^ However, completing the survey virtually or in the offered languages may have been challenging to some eligible participants and affected participation. Additionally, nonresponse bias remains a concern, as respondents may differ in their perspectives or experiences from those who did not participate, which could affect the generalizability of our findings. Overall, the large sample size of respondents, representing over 20 specialties, enforces our confidence in the generalizability of our findings. Although the multivariable analysis revealed statistically significant differences, the true percentage differences were small and may not represent clinically meaningful differences. Moreover, the survey, by design, only captures the perspectives of physicians and does not shed insight on the broader, multidisciplinary team perspective or those of childhood cancer patients and their families. To mitigate this limitation, nonphysician multidisciplinary team members and nonclinical advocates were intentionally involved in the preparatory dialogues at a regional and country level. Finally, future efforts should explore the perspectives of the health care and national program implementation teams to ensure success of design and implementation of interventions aimed at improving PC access.

In conclusion, this study identifies discrepancies between ideal and actual timing of PC integration in the care of children with cancer across AP. Physicians recognize early involvement of PC is vital for high-quality patient care and describe several barriers to the provision of PC in childhood cancer, including lack of resources, personnel, and PC-focused national policy and advocacy efforts. Although common challenges exist across the region, important differences are described between lower- and higher- resourced settings. Targeted strategies considerate of local contexts focused on equitable care delivery can help avoid widening existing health care access disparities within the region. Study results will be used to design interventions to improve PC capacity building, education, resource mobilization, and advocacy efforts. These initiatives will harness regional collaboration and collective experience from the PC community globally, ultimately improving PC and childhood cancer care across the region.

## Data Availability

Deidentified data are available upon request from authors.
